# Timing of Blood Sample Processing Affects the Transcriptomic and Epigenomic Profiles in CD4^+^ T-cells of Atopic Subjects

**DOI:** 10.3390/cells11192958

**Published:** 2022-09-22

**Authors:** Fahd Alhamdan, Kristina Laubhahn, Christine Happle, Anika Habener, Adan C. Jirmo, Clemens Thölken, Raffaele Conca, Ho-Ryun Chung, Gesine Hansen, Daniel P. Potaczek, Bianca Schaub, Ruth Grychtol, Holger Garn

**Affiliations:** 1 Translational Inflammation Research Division & Core Facility for Single Cell Multiomics, Medical Faculty, Philipps University of Marburg, Member of the German Center for Lung Research (DZL) and the Universities of Giessen and Marburg Lung Center, D-35043 Marburg, Germany; 2 Department of Medicine, Pulmonary and Critical Care Medicine, Brigham and Women’s Hospital, Harvard Medical School, Boston, MA 02115, USA; 3 Pediatric Allergology, Department of Pediatrics, Dr. von Hauner Children’s Hospital, University Hospital, Ludwig Maximilian University (LMU) of Munich, Comprehensive Pneumology Center Munich (CPC-M), Member of the German Center for Lung Research (DZL), D-80337 Munich, Germany; 4 Department of Pediatric Pneumology, Allergology and Neonatology, Hannover Medical School, Biomedical Research in Endstage and Obstructive Lung Disease Hannover (BREATH), Member of the German Center for Lung Research (DZL) and the Cluster of Excellence RESIST (EXC 2155), D-30625 Hannover, Germany; 5 Institute of Medical Bioinformatics and Biostatistics, Medical Faculty, Philipps University of Marburg, D-35032 Marburg, Germany; 6 Bioscientia MVZ Labor Mittelhessen GmbH, D-35394 Gießen, Germany

**Keywords:** CD4^+^ T-cells, transcriptomics, ChIP sequencing, histone modifications, epigenetics

## Abstract

Optimal pre-analytical conditions for blood sample processing and isolation of selected cell populations for subsequent transcriptomic and epigenomic studies are required to obtain robust and reproducible results. This pilot study was conducted to investigate the potential effects of timing of CD4^+^ T-cell processing from peripheral blood of atopic and non-atopic adults on their transcriptomic and epigenetic profiles. Two heparinized blood samples were drawn from each of three atopic and three healthy individuals. For each individual, CD4^+^ T-cells were isolated from the first blood sample within 2 h (immediate) or from the second blood sample after 24 h storage (delayed). RNA sequencing (RNA-Seq) and histone H3K27 acetylation chromatin immunoprecipitation sequencing (ChIP-Seq) analyses were performed. A multiplicity of genes was shown to be differentially expressed in immediately processed CD4^+^ T-cells from atopic versus healthy subjects. These differences disappeared when comparing delayed processed cells due to a drastic change in expression levels of atopy-related genes in delayed processed CD4^+^ T-cells from atopic donors. This finding was further validated on the epigenomic level by examining H3K27 acetylation profiles. In contrast, transcriptomic and epigenomic profiles of blood CD4^+^ T-cells of healthy donors remained rather unaffected. Taken together, for successful transcriptomics and epigenomics studies, detailed standard operation procedures developed on the basis of samples from both healthy and disease conditions are implicitly recommended.

## 1. Introduction

In recent years there has been a growing interest in diverse “omics” analyses utilizing different high-throughput techniques such as RNA sequencing (RNA-Seq) and chromatin immunoprecipitation sequencing (ChIP-Seq) [1,2]. These techniques have been employed to develop a better understanding of underlying molecular mechanisms and the pathobiology of a wealth of disease conditions including atopy [3,4,5]. The latter involves an increased risk of sensitization associated with the generation of inflammatory immune responses to harmless substances, based on a genetic predisposition to develop allergic diseases such as allergic rhinitis and asthma and/or mostly modern lifestyle-associated environmental influences [6,7,8].

Peripheral blood provides a minimally invasive liquid biopsy that comprises a vital source of molecular information and thus serves as an easily accessible diagnostic tool [9]. CD4^+^ T-cells in the blood mediate different adaptive immune responses to environmental structures including pathogens and allergens. Central to their function is the activation of other immune and non-immune cells by secreting many types of cytokines [10].

Various ways of handling peripheral blood samples during collection, shipping and processing prior to downstream analyses have a significant impact on the robustness and reproducibility of the results [11,12]. Processing of preselected cells at a given time point following blood collection in biobank sampling for big cohort studies represents logistical challenges that might overwhelm the subsequent biological associations [13].

In this pilot study, we intended to better define optimal experimental conditions for subsequent larger cohort studies by investigating potential impacts of timing of CD4^+^ T-cell processing from peripheral blood of atopic and non-atopic (healthy) adults on their transcriptomic (RNA-Seq) profiles. Moreover, we validated the RNA-Seq results on the epigenomic level by examining histone H3K27 acetylation (H3K27ac) profiles, known to be associated with active gene transcription, by ChIP-Seq [14]. 

## 2. Materials and Methods

### 2.1. Study Population and CD4^+^ T-Cell Sorting

**Figure 1:**
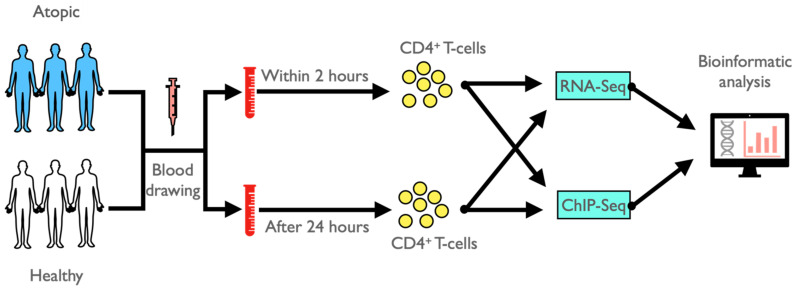
Schematic representation of the general study design.

In this pilot study, we recruited three atopic and three non-atopic (healthy) adult donors from two research centers of the German Center for Lung Research (DZL): two atopic and one healthy subject from the Department of Pediatric Pneumology, Allergology and Neonatology, Hannover and two healthy and one atopic subject from the Pediatric Allergology, Department of Pediatrics, Munich. All atopic subjects were diagnosed with sensitization to house dust mite, with either a history of hayfever (2 patients) or allergic rhinitis (1 patient). The patients were symptom-free and did not receive anti-inflammatory treatment at the time of analysis. Two heparinized blood samples were drawn from each of the six study subjects. The first blood sample was processed and CD4^+^ T-cells were FACS-sorted within two hours upon blood withdrawal (immediate processing), while the second blood sample was kept at room temperature (according to common protocols in biobank sampling) for 24 h prior to CD4^+^ T-cell isolation (delayed processing), followed by performing RNA-Seq and histone H3K27 acetylation ChIP-Seq applications and conducting bioinformatic analysis (Figure 1). In detail, heparinized venous blood was collected from each of the three atopic and healthy volunteers. Of each sample, 10 mL were immediately processed and 10 mL were left at room temperature for 24 h for delayed processing. At the two time points, peripheral blood mononuclear cells (PBMCs) were isolated by Biocoll (Biochrom, Berlin, Germany) density-gradient centrifugation and stained with fluorescent-labeled antibodies: CD8-FITC (RPA-T8), CD4-APC-H7 (RPA-T4) (Becton Dickinson, Heidelberg, Germany), CD19-PerCP-Cy5.5 (HIB19) (Biolegend, San Diego, CA, USA) and CD3-PE-Cy7 (UCHT1) (eBioscience, San Diego, CA, USA). CD3^+^CD4^+^CD8^−^CD19^−^ T-cells were sorted by fluorescent-activated cell sorting (FACS) using a FACSAria cell sorter (Becton Dickinson). The underlying gating strategy is shown in Figure S1. CD4^+^ T-cells were not cryopreserved at any stage of the analytical process. About 400,000 CD4^+^ T-cells were pelleted and re-suspended in RLT-Buffer (Qiagen, Hilden, Germany) including 1% β-Mercaptoethanol (Carl Roth, Karlsruhe, Germany) for transcriptome analysis and immediately transferred to −80 °C for storage. For ChIP-Seq analysis, about 600,000 CD4^+^ T-cells were washed and fixed in 1% formaldehyde solution (Merck, Darmstadt, Germany) followed by an addition of 1.3 M Glycine (Carl Roth) to crosslink DNA-associated proteins. Cells were washed once with ice-cold Dulbecco’s phosphate-buffered saline (pH 7.2) containing 1x protease inhibitor mix (Diagenode, Seraing, Belgium) and pelleted. After supernatant aspiration, pellets were immediately snap-frozen in liquid nitrogen and stored at −80 °C.

### 2.2. RNA Extraction, Library Preparation, and RNA Sequencing (RNA-Seq) Analysis

Total RNA was extracted from CD4^+^ T-cells using miRNeasy Mini Kit (Qiagen) according to the manufacturer’s protocol. RNA quality was assessed by Agilent Bioanalyzer 2100 (Agilent Technologies, Santa Clara, CA, USA). Library preparation was conducted using QuantSeq 3’ mRNA-Seq Library Prep Kit REV for Illumina (Lexogen, Greenland, NH, USA) according to the manufacturer’s protocol. Sequencing was performed on an Illumina NextSeq550 platform, High Output Kit v2.5, 50 bases single-reads (Illumina, San Diego, CA, USA) according to the manufacturer’s instructions.

### 2.3. Chromatin Immunoprecipitation Sequencing (ChIP-Seq) Analysis

ChIP-Seq analysis was performed using the True MicroChIP kit (Diagenode). Briefly, 100,000 fixed CD4^+^ T-cells were lysed and sheared using the Bioruptor Pico sonication device (Diagenode) for 15 cycles using a 30” [ON] 30” [OFF] setting. A total of 10% of the sheared chromatin was kept aside as an input control. For immunoprecipitation, sheared chromatin was incubated with 1 µg of an anti-H3K27ac polyclonal rabbit antibody. For quality check purposes, sheared chromatin was also incubated with 1 µg of unspecific rabbit IgG as isotype control (both antibodies from Diagenode). Samples were incubated with Protein A-coated magnetic beads and washed 4 times. Afterwards, the immunoprecipitated chromatin was de-crosslinked and DNA was purified. Quality check was performed for all samples by quantitative polymerase chain reaction (qPCR) using primer pairs (Diagenode) identifying a positive region, i.e., house-keeping gene *GAPDH* transcription starting site (TSS) control region, and myoglobin exon 2 as a negative control region using the Rotor-Gene SYBR Green PCR Kit (Qiagen). Afterwards, library preparation for H3K27ac and input control samples was performed using the MicroPlex Library Preparation Kit v3 with dual indexes (Diagenode) according to the manufacturer’s protocol. Sequencing was performed as described above.

### 2.4. Bioinformatic Analysis

RNA-Seq and ChIP-Seq bioinformatics workflows were performed using the Galaxy platform [15]. For RNA-Seq, quality of reads was assessed by FastQC v0.72 and reads were aligned to the human reference genome (Homo sapiens hg38 full) with RNA STAR v2.7.8a. Mapped reads were counted with featureCounts v2.0.1, followed by exon DESeq2 v2.11.40.6 analysis to determine differentially expressed genes. Enrichr BioPlanet and Gene ontologies databases were utilized to perform functional pathway enrichment analyses and investigate biological processes, respectively [16,17,18]. Gene expression values in Heatmaps were calculated as z-scores. ChIP-Seq reads were aligned to the same human reference genome using Bowtie2 v2.4.2. deepTools was utilized to generate enriched score genomic heatmaps and overlapped genes profiles. Differential whole gene analysis was conducted by DESeq2. MACS2 callpeak v2.2.7.1 and DiffBind v2.10.0 were used to perform peak calling and differential binding analyses, respectively. 

## 3. Results

### 3.1. Comparison of Transcriptome Profiles of Immediate Versus Delayed Processed CD4^+^ T-Cells from Atopic and Healthy Subjects

**Figure 2:**
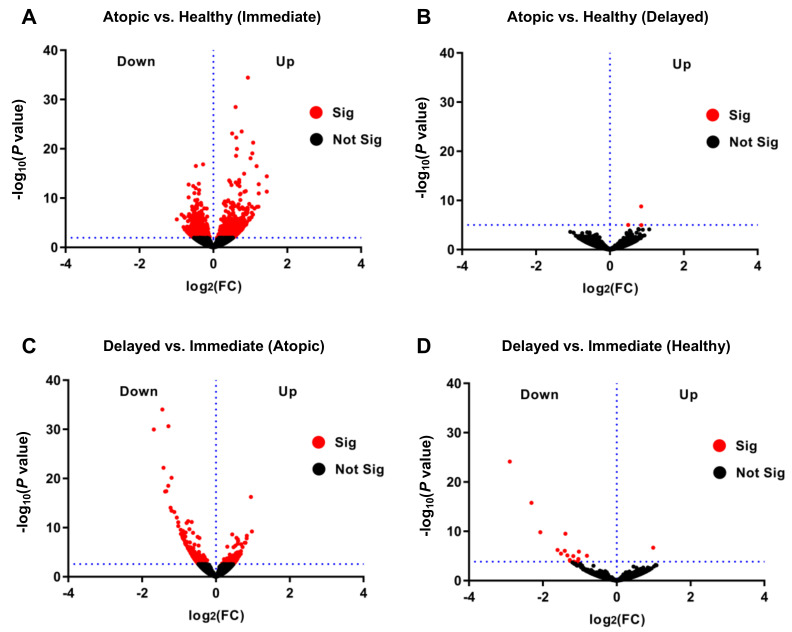
Differential transcriptome profiles in immediately versus delayed processed CD4^+^ T-cells from atopic and healthy subjects. Volcano plots presenting differentially regulated genes of (**A**) immediately and (**B**) delayed processed blood-derived CD4^+^ T-cells of atopic versus healthy subjects, and delayed versus immediately processed cells of (**C**) atopic subjects and (**D**) healthy subjects, all with significance threshold at FDR < 0.1.

**Figure 3:**
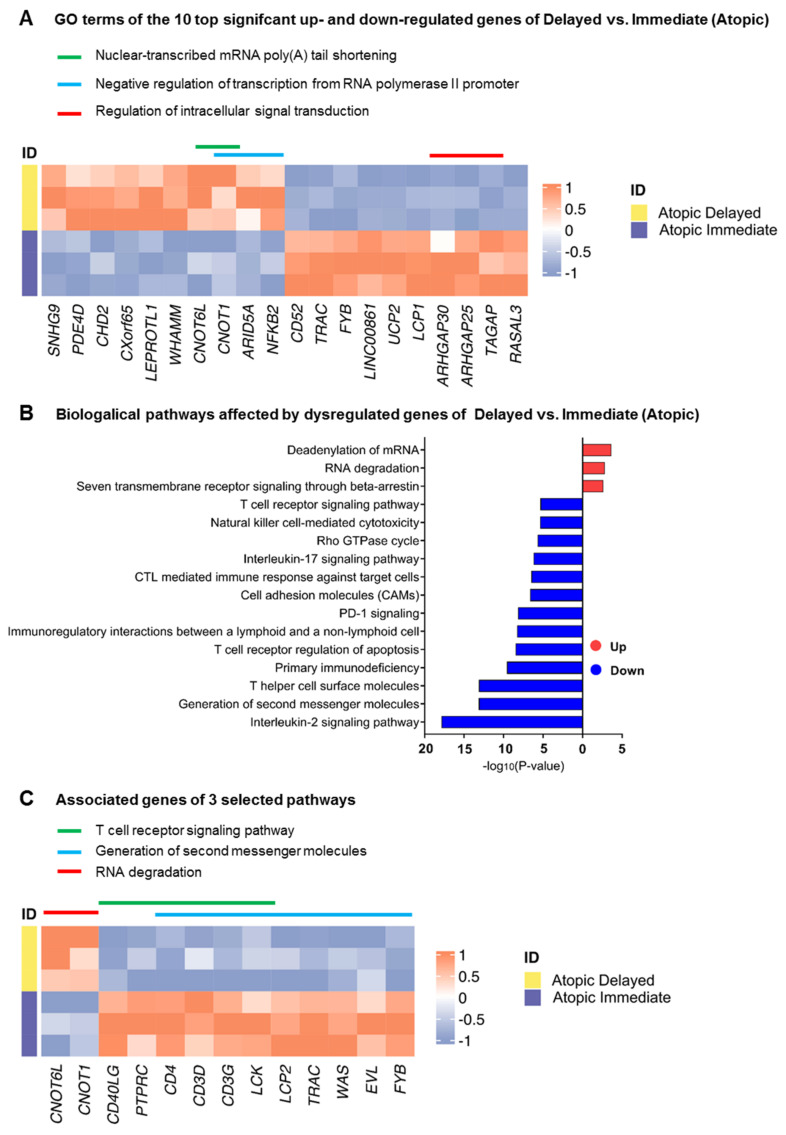
Biological processes and pathways affected by the differentially expressed genes in immediately processed CD4^+^ T-cells from atopic versus healthy subjects. (**A**) Heatmap depicting the top 10 significant up- and down-regulated genes of delayed versus immediately processed CD4^+^ T-cells from atopic subjects alongside their associated biological processes (GO Terms). (**B**) Biological pathways affected by up- and down-regulated genes of the previous comparison using a significance cut-off of *p* < 0.05, and (**C**) heatmap showing three selected biological pathways (indicated by colored lines) and their associated genes.

When comparing transcriptomic profiles of immediately processed CD4^+^ T-cells of atopic versus healthy subjects, a total of 1303 genes were identified to be differentially expressed at a false discovery rate (FDR) < 0.1 between these two groups (Figure 2A). We observed a multiplicity of genes involved in biological pathways known to play a role in atopy-associated T-cell activation processes (Figure S2A–C). Surprisingly, when performing the same analysis in delayed processed CD4^+^ T-cells, only three genes (*RPS27*, *RPL41*, and *RPL13*), all coding for ribosomal proteins, remained differentially expressed (Figure 2B). We then intended to explore the changes in the transcriptomes of the samples from the two time points of the same physiological condition. The above-described difference observed between delayed and immediate cell processing was predominantly caused by transcriptomic changes in CD4^+^ T-cells from atopic subjects since 320 genes were significantly differentially expressed when comparing immediately and delayed processed blood samples from atopic donors (Figure 2C). In contrast, the same comparison yielded only 17 differentially expressed genes in cells from healthy subjects (Figure 2D). Of note, expression of genes involved in deadenylation and degradation of mRNAs such as CCR4-NOT Transcription Complex Subunit 6 Like (*CNOT6L*) and CCR4-NOT Transcription Complex Subunit 1 (*CNOT1*) were found to be significantly up-regulated in delayed cell preparations from atopic subjects. In accordance, only pathways involved in the regulation of RNA stability were found to be upregulated in cells after delayed processing while a variety of atopy-related T-cell activation pathways (e.g., T helper cell surface molecules, T-cell receptor and IL-2 signaling pathways) were significantly down-regulated upon delayed processing (Figure 3A–C).

### 3.2. Epigenetic Profiles at the Level of Histone H3K27 Acetylation of Immediate Versus Delayed Processed CD4^+^ T-Cells from Atopic and Healthy Subjects

**Figure 4:**
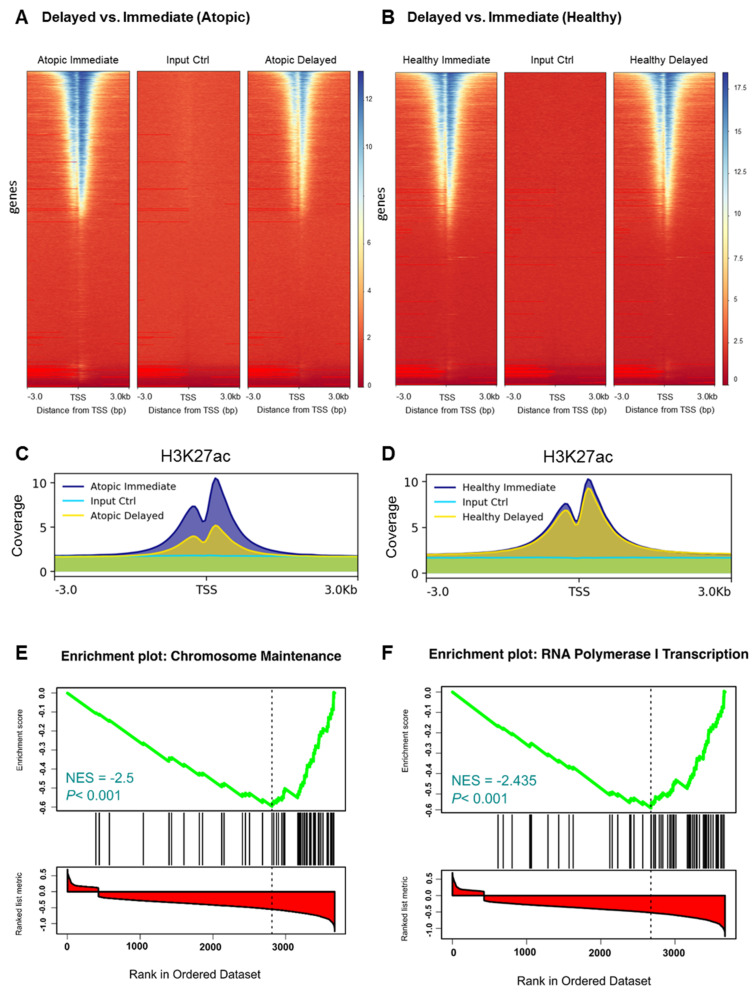
Selective histone modification changes within immediately versus delayed processed CD4^+^ T-cells from atopic subjects. (**A**,**B**) Genomic heatmaps visualizing H3K27 acetylation ChIP-Seq analyses scores associated with genomic regions of 3 kilobases (kb) around the gene transcription start site (TSS) and (**C,D**) average enrichments over all genomic regions of immediately and delayed processed blood-derived CD4^+^ T-cells from atopic (**A**,**C**) and healthy (**B**,**D**) subjects. (**E**,**F**) Gene set enrichment analysis (GSEA) of downregulated genes in delayed versus immediately processed blood-derived CD4^+^ T-cells from atopic subjects.

**Figure 5:**
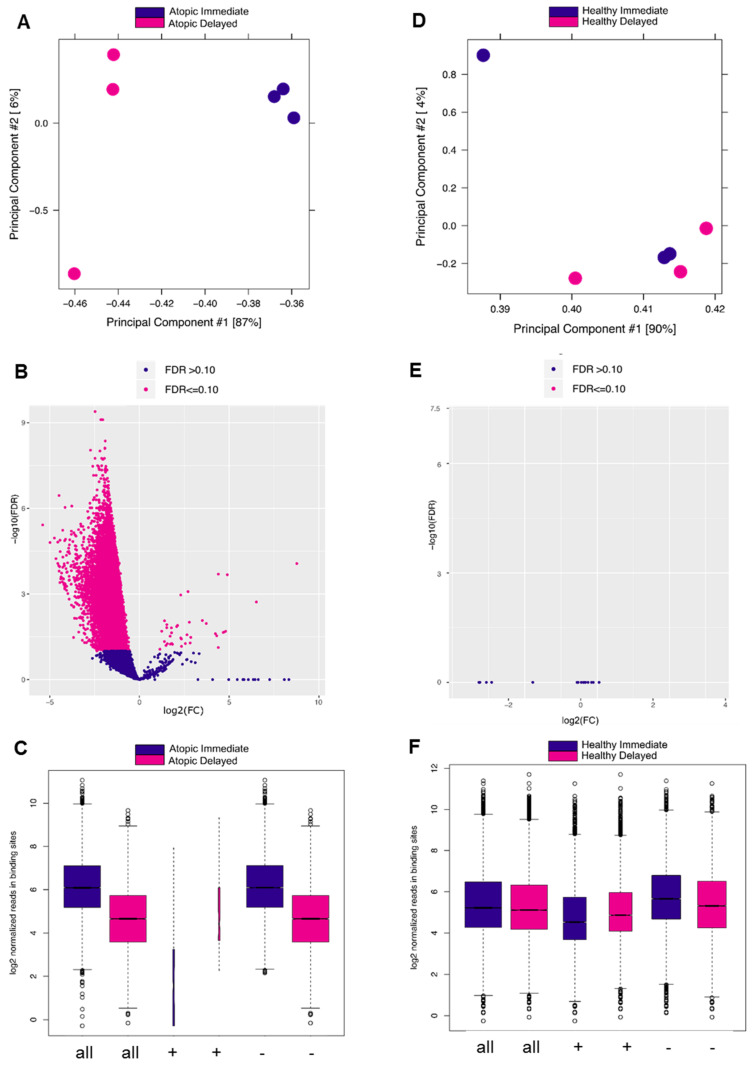
DiffBind analysis of called peaks of H3K27 acetylation ChIP-Seq analysis in delayed versus immediately processed blood-derived CD4^+^ T-cells of atopic and healthy subjects. (**A**) PCA plot showing the distance between samples based on the called peaks of delayed vs. immediate (atopic) comparison. (**B**) Volcano plot showing the differentially called peaks of the previous comparison (significance cut-off at FDR < 0.1). (**C**) Box plots of read distributions for significantly differentially called peaks of the previous comparison (binding affinity) for all sites (all), sites with increased affinity in the atopic delayed (+), and sites with increased affinity the atopic immediate (−) condition. (**D**–**F**) This is the same as the previous description for delayed vs. immediate (healthy) comparison.

To verify the previous findings on the epigenetic level, we carried out ChIP-Seq analysis of H3K27 acetylation, an epigenetic mark strongly correlating with active gene transcription [14]. When comparing delayed versus immediate cell processing, acetylation levels of H3K27 flanking around the TSSs were remarkably confined in the delayed processed CD4^+^ T-cells of atopic subjects (Figure 4A). In contrast, acetylation of H3K27 remained at comparable levels between the two processing time points in healthy donors (Figure 4B). To comprehensively demonstrate the different changes in the H3K27ac profiles, we combined the coverage of the H3K27ac histone marks at both time points and for both conditions (Figure 4C, D). Generally, these results confirmed the observations of the transcriptome analyses. In addition, we performed differential analysis on the genes encompassed by H3K27ac histone signals and obtained concordant results (Figure S3A,B). Biological pathway analysis of the differentially acetylated genes in the delayed versus immediate cell processing comparison of atopics revealed a variety of pathways affected by downregulated genes (Table 1) of which T-cell receptor and IL 2 signaling pathways were shared between the transcriptomic and epigenomic profiles. Additionally, ribosomal gene transcription and cell cycle-related pathways such as RNA polymerase I transcription and chromosome maintenance, respectively, were diminished during the 24 h ex vivo blood storage (Figure 4E,F). To verify the previous results at the level of H3K27ac peaks, we conducted a differential analysis of the called peaks using the DiffBind analysis tool. As a result, samples from atopic subjects segregated into two clusters demonstrating a discrete separation between the delayed and immediate time points of CD4^+^ T-cells processing (Figure 5A). The related volcano plot showed a greater gain of peaks in immediately processed samples compared to those prepared at the later time point (Figure 5B). Accordingly, the binding affinity analysis showed a higher mean read concentration to be related with the immediate time point (Figure 5C). On the other side, a similar analysis was applied to the healthy samples of both time points of CD4^+^ T-cells processing, yet no significant differences were observed in all parts of the analysis as shown in Figure 5D–F. 

## 4. Discussion

Pre-analytical processes represent a critical step in clinical biomaterial sampling [19]. Therefore, controlling and standardizing as many variables as possible in the experimental design of translational clinical and cohort studies is a critical prerequisite to achieve most reliable outcomes. Experimental pitfalls including timing of blood cell processing in biobank sampling are often not sufficiently considered [20,21]. In the pilot study presented here, we focused on atopy as a target disease condition that involves various activated immune mechanisms and is associated with prominent inflammatory characteristics [22,23]. CD4^+^ T-cells were selected for this study as a major cell type playing a key role in the underlying adaptive immune response [24].

The most striking result emerging from our data is not only that significant differences in gene expression were observed as a consequence of the ex vivo 24 h rest of blood samples, but also that such changes occurred quite specifically in CD4^+^ T-cells from atopic donors. Histone acetylation profiles and downstream gene expression associated with atopy was changed in CD4^+^ T cells following delayed processing. This resulted in the disappearance of significant differences observed in immediately processed CD4^+^ T-cells derived from atopic versus healthy individuals. This remark was supported by the downregulation of a variety of well-known atopy-related genes and their associated pathways such as IL-2, IL-17 and T cell receptor signaling pathways after 24 h storage of blood samples of atopic individuals [25]. Although too few studies have been conducted to investigate such pre-analytical effects, our results corroborated very well with observations by Baechler et al. [26]. In their study they utilized PBMCs of rheumatoid arthritis and systemic lupus erythematosus patients and observed time-dependent expression alterations in genes involved in inflammatory response, immune signaling and others. The emergence of RNA degradation genes and pathways after delayed CD4^+^ T-cells processing could propose evidence for the previous results [27,28]. However, the overall RNA quality did not vary much among all samples. This implies that key posttranscriptional control mechanisms/pathways involved in cell homeostasis are specifically triggered in CD4^+^ T-cells from atopic subjects upon delayed processing, presumably due to discontinuation of signals required for the maintenance of cellular activation during ex vivo blood storage [29]. However, it remains unclear why and how products of RNA degradation genes such as *CNOT1* and *CNOT6L* became specifically upregulated and how such mechanisms were selectively directed against atopy-related mRNA transcripts.

In addition, we investigated the epigenetic landscape as an upstream regulation mechanism of gene expression known to be involved in different allergic and atopic diseases [30]. Concordant results between the transcriptomic and epigenomic profiles were obtained, including a strong decrease in H3K27 acetylation signals spanning 3 kilobases (kb) around the TSSs in delayed processed CD4^+^ T-cells of atopics. These reduced histone acetylation signals were associated with genes involved in several molecular pathways (see Table 1), among them a variety of pathways involved in the regulation of general molecular and cellular processes such as control of gene expression, e.g., RNA polymerase I transcription or chromosome maintenance pathways [31].

Certainly, our work has some limitations such as small sample size and few time points of cell processing. Nevertheless, it outperformed previous studies in which similar timing effects were interrogated on other cell types derived from healthy donors only [32].

Taken together, our work demonstrates that delayed processing of blood-derived CD4^+^ T-cells has drastically changed their transcriptomic and epigenomic profiles specifically in atopic patients, which remained widely unaffected in healthy donors. RNA degradation mechanisms were activated in cells from atopic donors during prolonged 24 h ex vivo blood storage. To circumvent these time and condition-dependent pre-analytical effects, it is highly recommended to develop and apply standard operation procedures (SOPs) and protocols established on the basis of biomaterial samples derived from both patients and healthy individuals.

## Figures and Tables

**Table 1:** Top significant 30 biological pathways affected by down-regulated genes in H3K27ac ChIP-Seq analysis of delayed versus immediately processed blood-derived CD4^+^ T-cells from atopic subjects.

Term	*p*-Value	FDR
Translation	3.36 × 10^−20^	3.04 × 10^−17^
Influenza viral RNA transcription and replication	6.97 × 10^−20^	3.16 × 10^−17^
Cytoplasmic ribosomal proteins	1.98 × 10^−19^	6.00 × 10^−17^
Systemic lupus erythematosus	5.34 × 10^−19^	1.21 × 10^−16^
Influenza infection	6.30 × 10^−17^	1.14 × 10^−14^
Packaging of telomere ends	3.26 × 10^−13^	4.93 × 10^−11^
RNA polymerase I promoter opening	4.53 × 10^−13^	5.87 × 10^−11^
Cap-dependent translation initiation	4.55 × 10^−12^	5.16 × 10^−10^
T cell receptor regulation of apoptosis	4.31 × 10^−11^	4.34 × 10^−09^
Activation of mRNA upon binding of the cap-binding complex and eIFs, and subsequent binding to 43S	6.91 × 10^−11^	6.27 × 10^−09^
Meiotic recombination	1.91 × 10^−10^	1.57 × 10^−08^
Protein metabolism	6.45 × 10^−10^	4.87 × 10^−08^
Amyloids	1.79 × 10^−09^	1.25 × 10^−07^
Telomere maintenance	2.18 × 10^−09^	1.41 × 10^−07^
Gene expression	2.99 × 10^−09^	1.81 × 10^−07^
Meiotic synapsis	6.98 × 10^−09^	3.76 × 10^−07^
RNA polymerase I transcription	7.46 × 10^−09^	3.76 × 10^−07^
Meiosis	7.46 × 10^−09^	3.76 × 10^−07^
Deposition of new CENP-A-containing nucleosomes at the centromere	2.34 × 10^−08^	1.12 × 10^−06^
Transcription	2.49 × 10^−08^	1.13 × 10^−06^
RNA polymerase I, RNA polymerase III, and mitochondrial transcription	4.82 × 10^−06^	2.04 × 10^−04^
Type II interferon signaling (interferon-gamma)	4.95 × 10^−06^	2.04 × 10^−04^
Chromosome maintenance	5.32 × 10^−06^	2.10 × 10^−04^
Interleukin-2 signaling pathway	1.52 × 10^−05^	5.73 × 10^−04^
Messenger RNA splicing: major pathway	1.95 × 10^−05^	7.09 × 10^−04^
Diurnally regulated genes with circadian orthologs	3.06 × 10^−05^	0.001069
Respiratory electron transport, ATP biosynthesis by chemiosmotic coupling, and heat production by uncoupling proteins	1.18 × 10^−04^	0.003957
p75 neurotrophin receptor signaling via NF-kB	7.34 × 10^−04^	0.023787
Clathrin derived vesicle budding	8.94 × 10^−04^	0.027101
mRNA stability regulation by proteins that bind AU-rich elements	8.96 × 10^−04^	0.027101

## Data Availability

The data presented in this study are openly available in Dryad at doi:10.5061/dryad.zw3r22890.

## Supplementary Materials

The following supporting information can be downloaded online at: https://www.mdpi.com/article/10.3390/cells11192958/s1, Figure S1: Gating strategy for sorting of CD3^+^CD4^+^ T-cells. Stepwise gating is exemplarily indicated from left to right showing plots of related samples from one atopic subject with immediate and delayed processing. The purity of the sorted CD3^+^CD4^+^ T-cells used for the analyses is shown in the last plot to the right; Figure S2: Biological processes and pathways affected by the differentially expressed genes in immediately processed CD4^+^ T-cells in atopic versus healthy. (A) Heatmap depicting the top 10 significant up- and down-regulated genes of immediately processed CD4^+^ T-cells in atopic versus healthy subjects alongside with their associated biological processes (GO Terms). (B) Biological pathways affected by up- and down-regulated genes of the previous comparison using a significance cut-off of *p* < 0.05, and (C) heatmap showing three selected biological pathways (indicated by colored lines) and their associated genes; Figure S3: Differential gene analysis of H3K27ac ChIP-Seq analysis in delayed versus immediately processed blood-derived CD4^+^ T-cells. Volcano plots depicting genes associated with differential H3K27 acetylation identified by ChIP-Seq analyses (significance cut-off at FDR < 0.1) of delayed versus immediately processed blood-derived CD4^+^ T-cells from (A) atopic and (B) healthy subjects.
