# Supplementary material for: Timing of Blood Sample Processing Affects the Transcriptomic and Epigenomic Profiles in CD4+ T-cells of Atopic Subjects
Source: Cells. 2022 Sep 22;11(19):2958. doi: 10.3390/cells11192958 (PMC9563434; doi:10.3390/cells11192958)
Supplement: Supplementary file 1 [file cells-11-02958-s001.zip › cells-1825855-supplementary.pdf]

ONLINE SUPPLEMENT

Timing of blood sample processing affects the transcriptomic and epigenomic profiles in CD4$^+$ T-cells of atopic subjects

Fahd Alhamdan [1,2], Kristina Laubhahn [3], Christine Happle [4], Anika Habener [4], Adan C. Jirmo [4], Clemens Thölken [5], Raffaele Conca [3], Ho-Ryun Chung [5], Gesine Hansen [4], Daniel P. Potaczek [1,6], Bianca Schaub [3], Ruth Grychtol [4] and Holger Garn [1,*]

[1] Translational Inflammation Research Division & Core Facility for Single Cell Multiomics, Medical Faculty, Philipps University of Marburg, Member of the German Center for Lung Research (DZL) and the Universities of Giessen and Marburg Lung Center, D-35043 Marburg, Germany

[2] Department of Medicine, Pulmonary and Critical Care Medicine, Brigham and Women's Hospital, Harvard Medical School, Boston, MA 02115, USA

[3] Pediatric Allergology, Department of Pediatrics, Dr. von Hauner Children's Hospital, University Hospital, Ludwig Maximilian University (LMU) of Munich, Comprehensive Pneumology Center Munich (CPC-M), Member of the German Center for Lung Research (DZL), D-80337 Munich, Germany

[4] Department of Pediatric Pneumology, Allergology and Neonatology, Hannover Medical School, Biomedical Research in Endstage and Obstructive Lung Disease Hannover (BREATH), Member of the German Center for Lung Research (DZL) and the Cluster of Excellence RESIST (EXC 2155), D-30625 Hannover, Germany

[5] Institute of Medical Bioinformatics and Biostatistics, Medical Faculty, Philipps University of Marburg, D-35032 Marburg, German

[6] Bioscientia MVZ Labor Mittelhessen GmbH, D-35394 Gießen, Germany

* Corresponding author address
Prof. Dr. Holger Garn
Philipps University of Marburg - Medical Faculty
Translational Inflammation Research Division & Core Facility for Single Cell Multiomics
Biochemical Pharmacological Center (BPC)
Karl-von-Frisch-Str. 2
D-35043 Marburg, Germany
Email: garn@staff.uni-marburg.de
Tel.: +49 6421 2866040

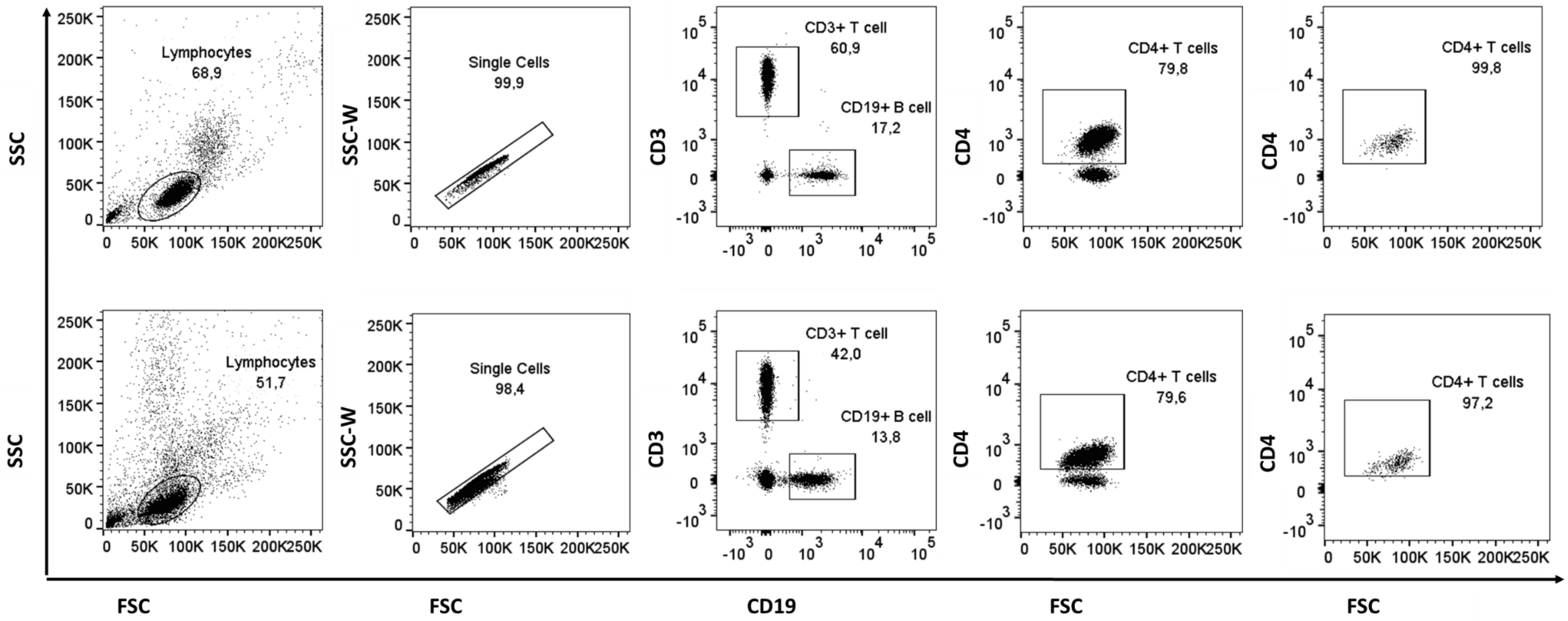

Figure S1. Gating strategy for sorting of CD3⁺CD4⁺ T-cells. Stepwise gating is exemplarily indicated from left to right showing plots of related samples from one atopic subject with immediate and delayed processing. The purity of the sorted CD3⁺CD4⁺ T-cells used for the analyses is shown in the last plot to the right.

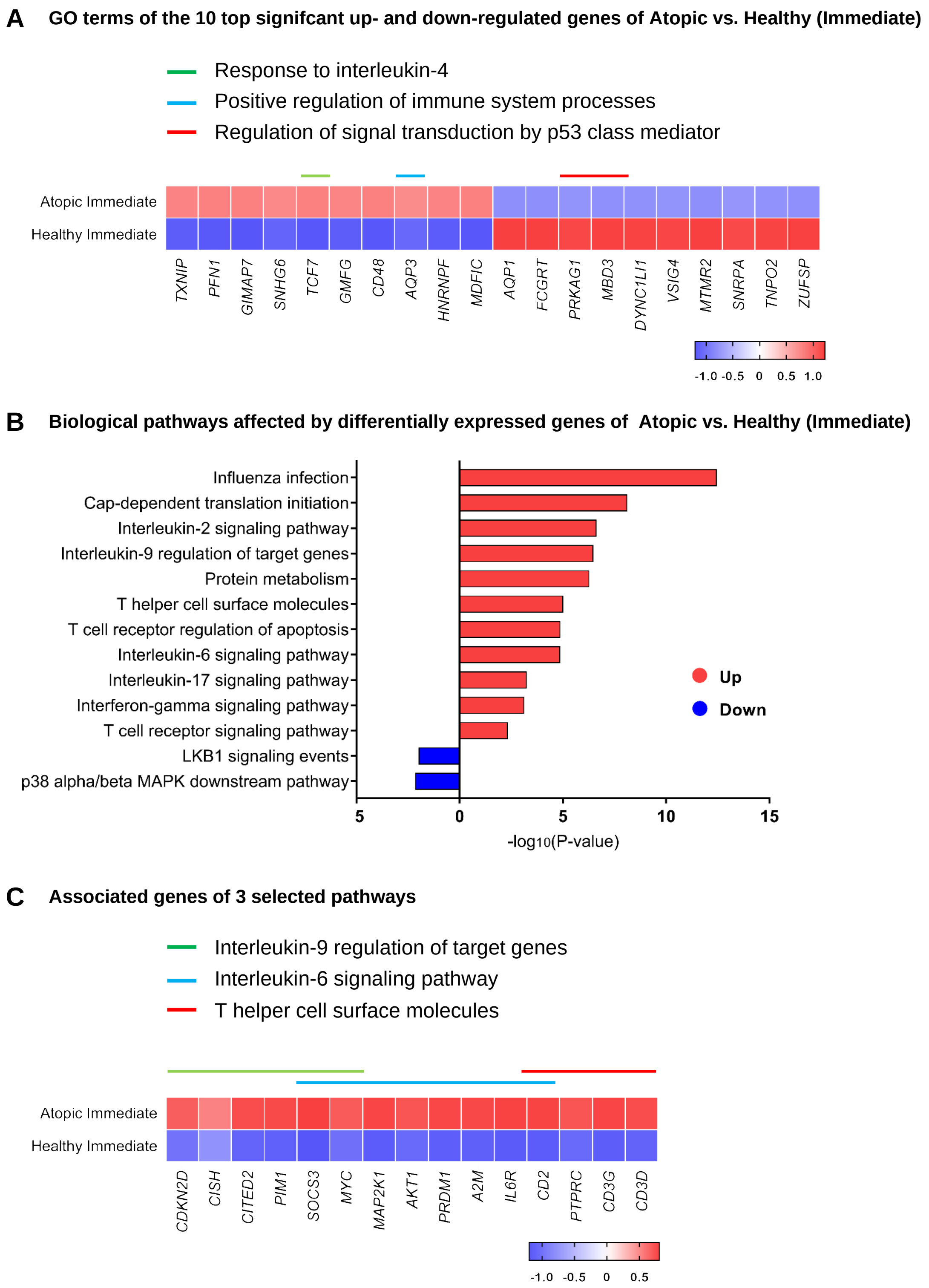

Figure S2. Biological processes and pathways affected by the differentially expressed genes in immediately processed CD4^{+} T-cells in atopic versus healthy. (A) Heatmap depicting the top 10 significant up- and down-regulated genes of immediately processed CD4^{+} T-cells in atopic versus healthy subjects alongside with their associated biological processes (GO Terms). **(B)** Biological pathways affected by up- and down-regulated genes of the previous comparison using a significance cut-off of $p < 0.05$, and **(C)** heatmap showing three selected biological pathways (indicated by colored lines) and their associated genes.

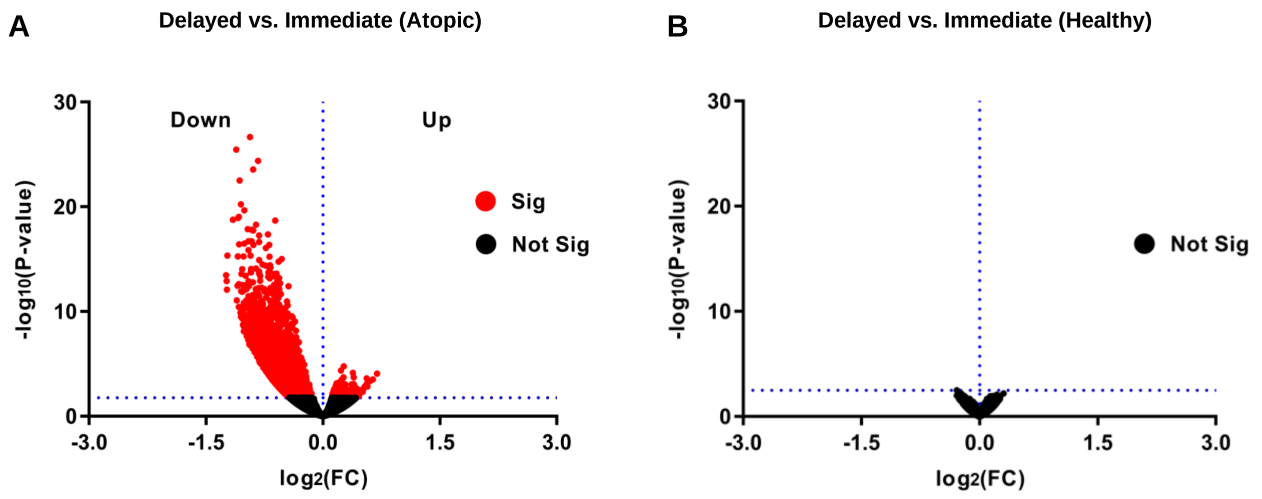

Figure S3. Differential gene analysis of H3K27ac ChIP-Seq analysis in delayed versus immediately processed blood-derived CD4$^+$ T-cells. Volcano plots depicting genes associated with differential H3K27 acetylation identified by ChIP-Seq analyses (significance cut-off at FDR < 0.1) of delayed versus immediately processed blood-derived CD4$^+$ T-cells from **(A)** atopic and **(B)** healthy subjects.
